# Myocardial Fibrosis in Young and Veteran Athletes: Evidence from a Systematic Review of the Current Literature

**DOI:** 10.3390/jcm13154536

**Published:** 2024-08-02

**Authors:** Richard P. Allwood, Michael Papadakis, Emmanuel Androulakis

**Affiliations:** 1 Sports Cardiology Department, Baker Heart and Diabetes Institute, Melbourne 3004, Australia; 2 Cardiovascular Clinical Academic Group, St George’s, University of London, London SW17 0RE, UK; mipapada@sgul.ac.uk

**Keywords:** cardiac magnetic resonance imaging, endurance athlete, late gadolinium enhancement, myocardial fibrosis, T1 mapping

## Abstract

**Background:** Exercise is associated with several cardiac adaptations that can enhance one’s cardiac output and allow one to sustain a higher level of oxygen demand for prolonged periods. However, adverse cardiac remodelling, such as myocardial fibrosis, has been identified in athletes engaging in long-term endurance exercise. Cardiac magnetic resonance (CMR) imaging is considered the noninvasive gold standard for its detection and quantification. This review seeks to highlight factors that contribute to the development of myocardial fibrosis in athletes and provide insights into the assessment and interpretation of myocardial fibrosis in athletes. **Methods:** A literature search was performed using the PubMed/Medline database and Google Scholar for publications that assessed myocardial fibrosis in athletes using CMR. **Results:** A total of 21 studies involving 1642 endurance athletes were included in the analysis, and myocardial fibrosis was found in 378 of 1595 athletes. A higher prevalence was seen in athletes with cardiac remodelling compared to control subjects (23.7 vs. 3.3%, *p* < 0.001). Similarly, we found that young endurance athletes had a significantly higher prevalence than veteran athletes (27.7 vs. 19.9%, *p* < 0.001), while male and female athletes were similar (19.7 vs. 16.4%, *p* = 0.207). Major myocardial fibrosis (nonischaemic and ischaemic patterns) was predominately observed in veteran athletes, particularly in males and infrequently in young athletes. The right ventricular insertion point was the most common fibrosis location, occurring in the majority of female (96%) and young athletes (84%). Myocardial native T1 values were significantly lower in athletes at 1.5 T (*p* < 0.001) and 3 T (*p* = 0.004), although they had similar extracellular volume values to those of control groups. **Conclusions:** The development of myocardial fibrosis in athletes appears to be a multifactorial process, with genetics, hormones, the exercise dose, and an adverse cardiovascular risk profile playing key roles. Major myocardial fibrosis is not a benign finding and warrants a comprehensive evaluation and follow-up regarding potential cardiac disease.

## 1. Introduction

Exercise is associated with several structural, functional, and electrical cardiac adaptations. These usually result in a balanced increase in the cardiac chamber size and myocardial mass, which is often referred to as athlete’s heart [1]. The magnitude of these adaptations is influenced by the athlete’s age, sex, ethnicity, body size, and sporting discipline, including the intensity, duration, and years of sports practice [1]. Often, the most profound effects are seen in male endurance athletes.

Emerging evidence, however, suggests that high-volume, high-intensity exercise training over time may be associated with adverse cardiac remodelling, such as myocardial fibrosis (MF) [2]. MF is characterised by fibroblast activation and collagen infiltration into the extracellular matrix, which can develop in response to injury from myocardial ischaemia, inflammation, or pressure overload [3]. MF is divided into reactive and replacement fibrosis. The reactive type is an earlier and reversible marker of myocardial disease, involving increased collagen synthesis in response to cardiac stress. Meanwhile, replacement or focal fibrosis is irreversible, involving collagen replacement following myocyte apoptosis or necrosis and resulting in myocardial scarring [4].

MF can be assessed by a variety of methods in clinical practice. Traditionally, endomyocardial biopsy, an invasive procedure, is used for determining the presence of MF and is regarded as the gold standard [3]. Other tools currently available to characterize MF include biomarkers and noninvasive imaging modalities, such as echocardiography and strain analysis, cardiac computed tomography, single-photon emission computed tomography and positron emission tomography, and CMR [3,4]. CMR provides detailed tissue characterization using methods that focus primarily on the composition of the extracellular space.

MF is assessed noninvasively using CMR imaging with gadolinium to determine late gadolinium enhancement (LGE), a marker of replacement MF. However, LGE typically represents only focal regions of increased fibrosis. Parametric T1 mapping, such as myocardial native T1 values and the extracardiac volume (ECV) can be used to assess myocardial composition. This emerging technique can detect focal or diffuse interstitial disease, although it has not been well established in the athlete population [5,6]. Late gadolinium enhancement CMR imaging has emerged as a powerful tool in the field of sports cardiology due to its unique ability to comprehensively assess myocardial structure and function. It has become increasingly useful for differentiating structural features in athletes that overlap with cardiomyopathies, providing important guidance to physicians regarding sports participation [7].

Interestingly, specific patterns of LGE have been observed more frequently in athletes, although these data are not always consistent and have not been compared to those of the general population or veteran athletes [2,5,7]. A pattern-based approach has been described for LGE assessment [8]. MF can be described as major or minor [9]. Major MF is defined as overt fibrosis in the compacted myocardium involving nonischaemic (mid-myocardial and subepicardial LGE) or ischaemic (subendocardial and transmural LGE) patterns [7]. Meanwhile, minor MF typically affects the anterior and inferior right ventricular (RV) insertion points (with a non-specific pattern in athletes), papillary muscles, or RV trabeculae [9].

MF is associated with increased myocardial stiffness, heart failure, a higher incidence of ventricular arrhythmias (VAs), and adverse cardiac outcomes such as sudden cardiac death (SCD) [2,3,9]. However, the aetiology and clinical implications of MF in athletes are still unclear. This review seeks to highlight the factors that contribute to the development of MF in athletes and assess its effects on cardiac structure and function.

## 2. Methods

**Figure 1:**
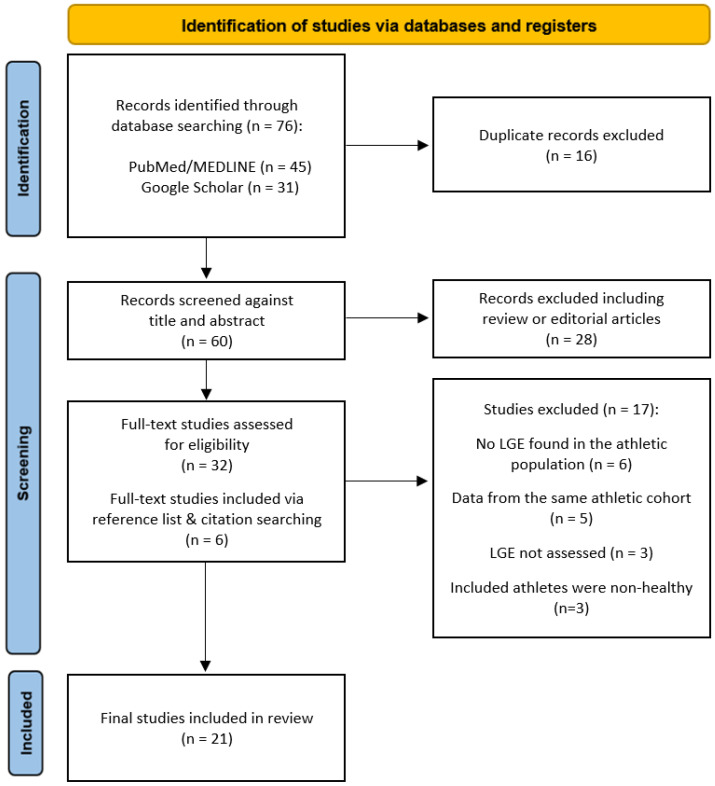
Flow chart of the literature selection.

A literature search was performed using the PubMed/Medline database and Google Scholar. This review was conducted in accordance with the Preferred Reporting Items for Systematic Reviews and Meta-Analyses (PRISMA) guidelines [10]. The search strategy was not limited by the date of publication and was restricted to the English language. Medical subject headings and free search terms were used individually and in combination. The terms included athlete AND athlete’s heart AND myocardial fibrosis AND fibrosis AND scar AND cardiac magnetic resonance imaging AND late gadolinium enhancement OR delayed gadolinium enhancement AND T1 mapping OR native T1 values OR extracellular volume quantification. In addition, other relevant publications were identified using a manual search of the reference and citation lists from all of the eligible studies (Figure 1).

Studies were included in which high-performance endurance athletes were assessed using CMR to determine the presence of MF. Outcomes of studies that evaluated one or more of the following parameters in athletes were eligible for inclusion: (a) the presence of late gadolinium enhancement, (b) native T1 values, and (c) extracellular volume. Endurance athletes were defined as having had competition experience or a long period of regular high-intensity endurance training [6]. A mean age of >40 years characterised veteran athletes [5]. Only studies reported in English were evaluated for inclusion. Publications based on athletes with known cardiovascular disease or included on the basis of having signs or symptoms of cardiac disease (e.g., premature ventricular beats) were excluded from the systematic review. When multiple studies reported results using the same cohort data from the same research group, only one was kept, unless no overlap was identified. A detailed presentation of the systematic protocol is described in Supplementary Materials. Quality and bias assessment was performed via the Newcastle–Ottawa quality assessment scale (NOS) for cohort studies (Supplementary Table S1). Between-group differences were assessed using Student’s *t*-tests, with a *p*-value < 0.05 considered statistically significant.

## 3. Results

The results of the literature search are displayed in Table 1 [11,12,13,14,15,16,17,18,19,20,21,22,23,24,25,26,27,28,29,30,31]. A risk of bias assessment was performed using the Newcastle–Ottawa Scale (Supplementary Table S1). Across 21 studies, there were 1642 athletes with a mean age of 43.36 ± 6.8 years (with a range of 15–82). Overall, 78% of the athletes were male. However, the age and sex of 40 athletes were not reported. The athletes were mostly long-term, highly trained endurance participants who engaged in high volumes of exercise per week (with a mean of 21.7 ± 9.2 years and 9.8 ± 3.8 h per week of training, based on 16 studies) [1]. The majority participated in running and cycling, competing in triathlons and marathons.

MF was reported in 21 studies, with 23.7% of the athletes (378 of 1595) and 3.3% of the controls (20 of 602, 15 studies) (*p* < 0.001) showing evidence of LGE. A higher prevalence was seen in young (27.7% of 775, eight studies) compared to veteran (19.9% of 820, 14 studies) athletes (*p* < 0.001). Meanwhile, 3.5% of the veteran controls (18 of 515, 13 studies) and 2.3% of the young controls (2 of 87, 2 studies) (*p* = 0.565) showed LGE.

In 16 studies in which sex was differentiated, LGE was found in 193 of 980 male athletes (19.7%) and 47 of 287 female athletes (16.4%) (*p* = 0.207). Athletes with MF showed greater cardiac remodelling, with larger heart chambers and a greater mass than athletes without MF [2,4,7,11,12,13,14,15,16,17,18,20].

**Figure 2:**
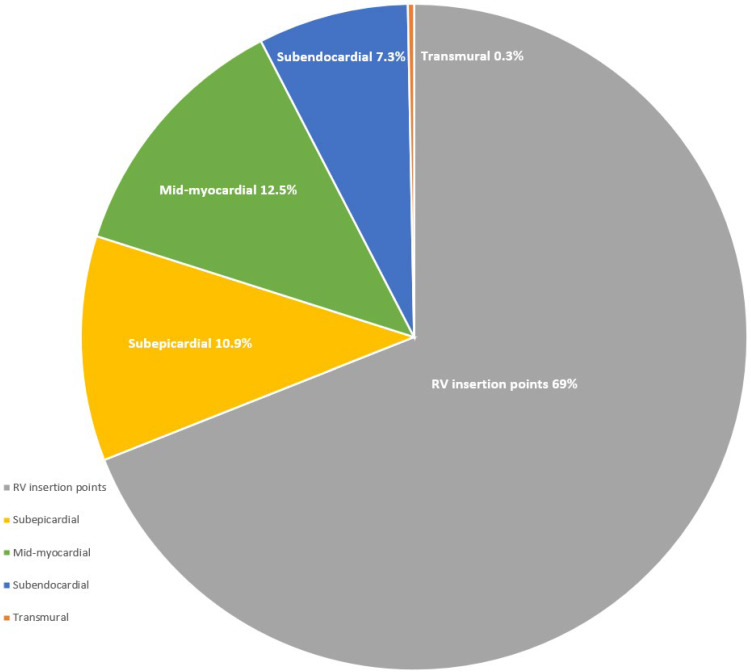
Patterns and locations of LGE in athletic populations consisting of major (nonischaemic in 23.4% and ischaemic in 7.6% of cases) and minor MF (RV insertion points in 69% of cases).

**Figure 3:**
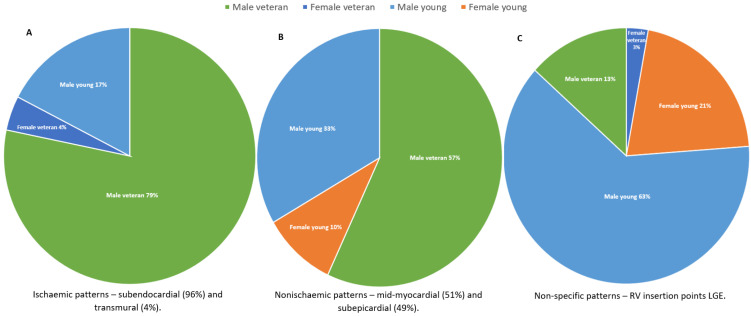
MF patterns and locations characterised by the athletes’ age and sex (**A**) Ischaemic patterns, (**B**) Nonischaemic patterns, (**C**) Non-specific patterns).

In 19 studies, LGE patterns were reported in 303 athletes (80% male), with major MF patterns found in 31% of cases, consisting of nonischaemic (23.4%) and ischaemic (7.6%) types (Figure 2). Major MF patterns were found significantly less frequently in young athletes (38%) compared to veteran athletes (62%) (*p* < 0.001). A nonischaemic pattern presented significantly more frequently in veteran athletes (57%) compared to young athletes (43%) (*p* = 0.008). Similarly, an ischaemic pattern was more common in veteran athletes (83%) compared to young athletes (17%) (*p* < 0.001) (Figure 3).

The most common location of LGE followed a non-specific pattern, such as the RV insertion point (69%). Interestingly, 96% of female (n = 49) and 84% young (n = 215) athletes had this pattern. This was significantly higher in young athletes compared to veterans (16%) (*p* < 0.001). Overall, veteran athletes showed nonischaemic patterns (43%), followed by non-specific (37%) and ischaemic (20%) patterns, while young athletes showed non-specific (84%), nonischaemic (14%), and ischaemic (2%) patterns.

Myocardial composition was further assessed using native T1 time and ECV quantification in nine studies (Table 2). Athletes had normal native T1 times, although statistically lower mean values at 1.5 T (990.09 ± 32 ms vs. 1029 ± 27 ms, *p* < 0.001) and 3 T (1185.8 ± 34 ms vs. 1207.5 ± 32 ms, *p* = 0.004) when compared to the control groups. There was no significant difference for mean myocardial ECV values at 1.5 T (26.2 ± 2.1% vs. 25.95 ± 2.9%, *p* = 0.432) and 3 T (23.2 ± 3.3% vs. 22.84 ± 2.6%, *p* = 0.366) between the athlete and control groups. Veteran athletes showed similar results, while female athletes had slightly higher ECV values. Those with LGE demonstrated slightly higher native T1 times and global or remote ECV values, although still within normal ranges [19].

## 4. Discussion

This is the largest systematic review on endurance athletes with MF, which aimed to highlight the prevalence of MF among athletes and provide insights into its assessment and interpretation. We found a significantly higher prevalence of MF in athletes with cardiac remodelling compared to control subjects, with specific patterns more commonly associated with endurance athletes’ age and sex. In this review, when compared to two previous meta-analyses [5,6], we found a similar prevalence of MF in endurance athletes compared to the meta-analysis by Zang et al. (21.1%) [6]; however, we observed a higher prevalence of non-specific (RV insertion point) patterns and a lower prevalence of major MF patterns (69% vs. 31%) in these endurance athletes compared to their review (31% vs. 69%) [6]. This difference is owed to the updated set of studies, which included new data involving greater numbers of high-level young endurance athletes.

A higher prevalence of LGE was seen between the young athletes compared to veteran athletes in this review, which was a similar finding in the meta-analysis by Androulakis et al. [5], in which young athletes had a higher incidence of LGE compared to veteran athletes (25.7% vs. 14.6%, *p* = 0.002). Androulakis et al. [5] showed that non-RV insertion point LGE prevalence was significantly higher in athletes compared to controls (7% vs. 0.2%, *p* = 0.003). In this review, this pattern was further evaluated, with nonischaemic and ischaemic patterns significantly more common in veteran athletes, particularly males, and infrequent in young athletes. Meanwhile, female and young athletes presented more often with a non-specific pattern, such as RV insertion point LGE. Interestingly, the athletes’ native T1 values were reduced compared to control groups, with similar ECV values which was a similar finding to that of Androulakis et al. [5].

### 4.1. Myocardial Fibrosis in Athletes and the General Population

Several studies have shown evidence of MF in high-level endurance athletes in 2 to 50% of small-scale athletic populations [13,18,20,24,30]. However, not all of these studies have demonstrated MF in athletic groups [32,33]. In a large general population study, MF was reported in 7.9% of patients (62% nonischaemic vs. 38% ischaemic scars), 78% of whom were not recognized in clinical evaluations or via electrocardiography [34]. A higher prevalence of undiagnosed MF (17% and 19.8%) was reported in other general population studies [35,36]. Interestingly, those with scars were more likely to be older, male, and smokers, with higher blood pressure, body mass index, and coronary calcium scores [34].

### 4.2. Factors Associated with Myocardial Fibrosis in Athletes

#### 4.2.1. Exercise Dose

Several studies have suggested a strong association between the volume of lifelong endurance exercise and the extent of MF [3,11,18,20,24]. Wilson et al. [20] found that six (50%) male veteran athletes demonstrated LGE, but none of the 17 young endurance athletes and 20 age-matched controls did. LGE was associated with the number of years spent training, as well as the number of completed competitive marathons and ultra-endurance (>50 miles) marathons.

Breuckmann et al. [24] demonstrated a higher prevalence of LGE in 102 male veteran marathon runners compared to age-matched controls (11.8% vs. 3.9%), with the number of marathons functioning as an independent predictor of the presence of LGE. Tahir et al. [11] also showed an association between MF and race distance in male triathletes, with LGE+ athletes completing longer cumulative distances and more middle and ironman distances than LGE- athletes. La Gerche et al. [18] found minor focal MF in five endurance athletes who had greater cumulative exercise exposure. The investigators also showed that intense endurance exercise can cause transient RV dilatation and dysfunction and elevated cardiac biomarkers (brain natriuretic peptide and cardiac troponin I) after endurance races. Recovery occurred in most athletes within one week. The study highlighted a key step in the potential pathophysiological development of MF in athletes. It linked repeated bouts of myocardial dysfunction to intense exercise, leading to adverse cardiac remodelling [37]. Evidence from animal models may also support this mechanism. Benito et al. [38] showed that in young male rats, endurance training demonstrated training-dependent RV MF and increased VA following 16 weeks of a chronic exercise regime. These findings were reversed after eight weeks of exercise cessation. However, most of these studies investigated male veteran athletes, with limited numbers of female athletes included. Interestingly, female athletes may have lower cumulative exposure to vigorous exercise compared to males, which may account for some of the differences in MF between male and female athletes [11,12,17].

#### 4.2.2. Pressure Overload

Pressure overload has also been proposed as a potential haemodynamic mechanism for the development of MF in athletes. Rises in ventricular wall stress with exercise and the duration of exercise stress have been suggested as important contributing factors [18,39]. Tahir et al. [11] showed that exercise-induced systolic hypertension was an independent predictor of MF. In 83 asymptomatic triathletes (training >10 h/w), 16.7% of males showed nonischaemic MF, but none of the female athletes did. Interestingly, LGE+ athletes had significantly higher peak levels of systolic exercise blood pressure (213 ± 24 mmHg vs. 194 ± 26 mmHg, *p* < 0.05), LV mass indices (93 ± 7 g/m^2^ vs. 84 ± 11 g/m^2^, *p* < 0.05), and ECV values (26.3 ± 1.8% vs. 24.4 ± 2.2%, *p* < 0.05) compared to LGE- athletes. However, female athletes demonstrated a lower peak exercise blood pressure and shorter race distances compared to male athletes [11]. In general, female athletes appear to have lower blood pressure at rest [40] and with exercise [41], which may be another reason for the difference in MF between male and female athletes.

Minor focal MF located in the RV insertion points is thought to occur due to RV pressure and/or volume overload, resulting from damage to myocytes [2,3]. In endurance athletes, exercise-induced elevations in pulmonary artery systolic pressure can occur. La Gerche et al. [39] demonstrated that RV wall stress increases 30-fold during exercise, reflecting a significant rise in pulmonary artery systolic pressure. This raises the possibility that repetitive, intense exercise may induce chronic structural changes in the RV insertion points in endurance athletes.

#### 4.2.3. Viral Myocarditis

Several studies have suggested that myocarditis may be responsible for major MF in athletes [2,3,42]. Tahir et al. [11] showed that LGE+ athletes exhibited a nonischaemic pattern in 55% of cases, which may be consistent with prior silent myocarditis involving the mid-myocardial and subepicardial locations. This pattern may also be associated with other conditions, such as arrhythmic, dilated, or hypertrophic cardiomyopathies [2]. However, small mid-wall areas of nonischaemic MF have also been observed in 4% of the general population [34] and seem to have no significant prognostic implications [43]. Viral infections have been linked to subclinical myocarditis, occurring more frequently in younger men than women, suggesting a strong relationship with testosterone levels [3,11,42]. Testosterone may promote an immune response that leads to inflammation and fibrosis, while oestrogen may result in less inflammation during myocarditis in females [44]. Using animal models, researchers found that physical activity during myocarditis worsened heart damage in male mice [45]. In athletes who continue to exercise during an infection, it is thought that this may weaken immune function, leading to myocardial dysfunction, arrhythmias, and MF [2,42].

#### 4.2.4. Coronary Artery Calcification

Some studies have suggested a link between the coronary plaque burden and myocardial damage in marathon runners [12,26,46]. Vigorous exercise at a very high intensity and increasing exercise volume may accelerate the progression of atherosclerotic disease, with possible links to vascular wall damage and exercise-induced metabolic or hormonal changes [12,46,47,48]. Möhlenkamp et al. [46] found that coronary artery calcification was higher in male veteran marathon runners with major MF (with 42% showing an ischaemic pattern). These athletes had low Framingham risk scores (7 ± 3.6%), despite some having a history of hypertension (12%) and smoking (51.9% former smokers and 4.6% active smokers). Coronary artery calcification and the frequency of marathon running were shown to be independent predictors of the presence of LGE [46]. Karlstedt et al. [26] proposed a similar pathophysiological link in marathon runners, with two male veteran marathon runners showing subendocardial LGE and obstructive coronary artery disease.

In contrast, a study conducted by Merghani et al. [12] showed no adverse cardiac remodelling in female veteran endurance athletes. A similar prevalence of coronary artery disease was observed in sedentary female controls. However, male veteran athletes had higher coronary artery calcification scores and evidence of major MF (14.2% vs. 0) and VA (9.4% vs. 0) compared to sedentary male controls. In these athletes, their age and years of endurance training were determinants of significant coronary artery disease [9,12] and a predictor of major MF [9] (with 56% having nonischaemic, 33% having ischaemic, and 11% having mixed patterns). Interestingly, in these three studies, an ischaemic pattern was present in 50% of LGE+ middle-aged veteran athletes (6.7% female) [12,26,46]. This pattern is the least common one observed in athletes, occurring with a similar frequency to that of sedentary individuals [2]. It has been suggested that genetics, a lower cardiovascular risk profile, and the protective effects of hormones such as oestrogen in females may play a role in preventing adverse cardiac remodelling, when compared to male veteran athletes [12,34,39,49].

The pathological consequences of MF can contribute to structural and electrical changes, leading to alterations in cardiac function and arrhythmias, particularly in those associated with major MF [2]. Excessive collagen deposition can disrupt the mechanical-electrical coupling of myocytes, predisposing athletes to VA and sudden death [3,50]. Fibrosis diffuse interstitial fibrosis widely throughout the myocardium, measured by ECV, has been shown to promote arrhythmias more often than focal MF detected by LGE, which typically involves only small portions of the myocardium [51].

Furthermore, interaction between diffuse MF and myocytes in perivascular areas can restrict the supply of oxygen and nutrients to the myocardium, further exacerbating adverse remodelling responses [2,50]. These structural changes can lead to adverse outcomes such as myocyte ischaemia, as well as decreased myocardial compliance and impaired contractility, leading to heart failure.

### 4.3. Parametric T1 Mapping

**Figure 4:**
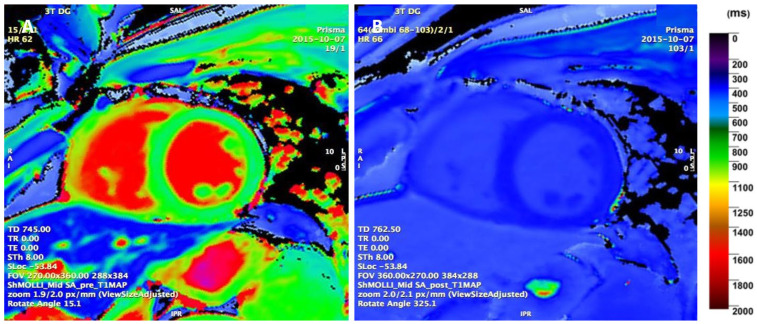
(**A**) T1 mapping with native T1 (1126 ± 46 ms) and (**B**) post-contrast T1 (490 ± 38 ms) images (ECV 22.9%) (ShMOLLI 3T) in a 24-year-old male runner.

T1 mapping can provide valuable information regarding physiological or pathological cardiac remodelling in athletes and earlier insight into pathological changes compared to LGE [2,5] (Figure 4). Native T1 and ECV values can increase in LGE+ athletes, suggesting the presence of diffuse interstitial fibrosis and extracellular expansion [11,19]. These values increase with the burden of fibrosis and show a good correlation with histologic evidence of interstitial MF [4,27,52]. Meanwhile, normal or lower values in athletes with a higher LV mass can represent physiological remodelling due to greater myocyte hypertrophy with no extracellular expansion [9,19,27,28]. To prevent the overdiagnosis of pathological findings, these mapping parameters should be assessed in the appropriate clinical context and should not be reported in isolation [53].

### 4.4. Clinical Implications

**Figure 5:**
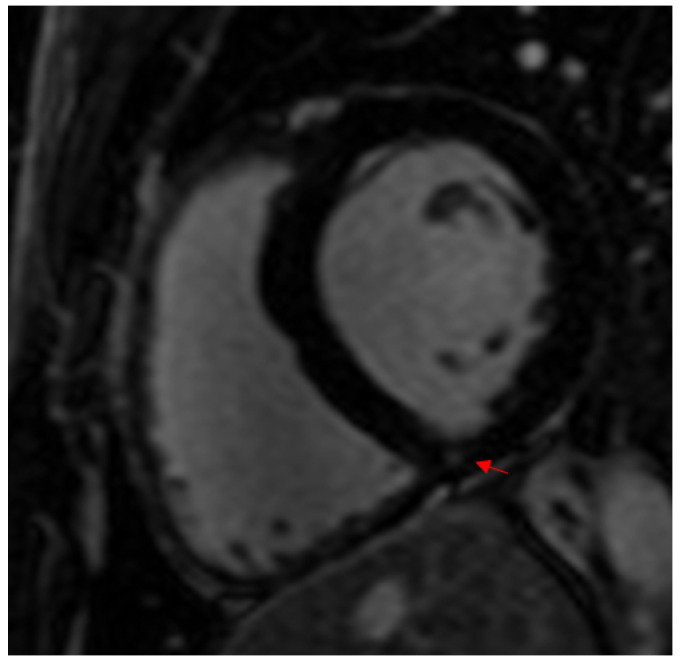
LGE of the inferior RV insertion point in a young male triathlete.

**Figure 6:**
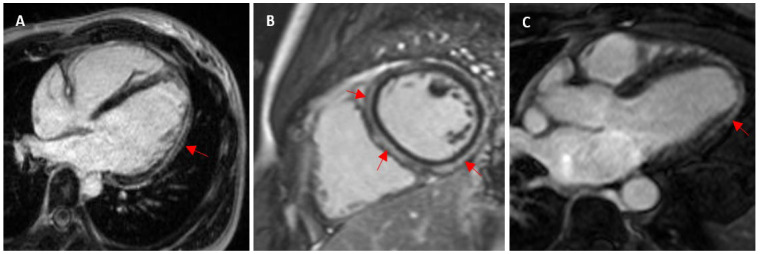
(**A**) Subepicardial scar in a 20-year-old female swimmer with VA (arrhythmogenic cardiomyopathy—gene elusive). (**B**) Mid-myocardial and subepicardial LGE in a 31-year-old male triathlete presenting with ventricular ectopy and chest pain (arrhythmogenic cardiomyopathy—desmin gene variant). (**C**) Mid-myocardial and subepicardial LGE in a 51-year-old male runner with previous reports of chest pain and raised inflammatory markers (prior myocarditis).

Irrespective of age and sex, a pattern of minor focal MF, particularly the RV insertion point location (Figure 5), is commonly seen in endurance athletes with cardiac remodelling [2,9,14,15,16,18,19,20,23]. This location of fibrosis has been correlated with the cumulative training load and intensity [15,16,18,19,20]. However, the volume of LGE is typically small and has also been found in similar locations in healthy elderly individuals and patients with pulmonary hypertension [2]. This non-specific pattern has been shown to be benign and is not associated with VA; thus, it may be considered an incidental finding [2,3,7,23,25,54]. However, further follow-up studies may be required to assess the long-term outcomes of this pattern. Several studies have emphasized the clinical importance of major MF, such as a nonischaemic pattern of LGE in athletes [31,55,56,57] (Figure 6). This pattern should not be considered a benign finding; it has been associated with VA, progressive LV dysfunction, and SCD. Zorzi et al. [56] studied 27 young athletes with a mid-myocardial and subepicardial stria of LGE, mostly involving the lateral LV wall, which was associated with malignant arrhythmic events, regional LV dysfunction, and SCD. Schnell et al. [58] followed seven asymptomatic young professional athletes (86% male) with extensive subepicardial LGE. The majority experienced non-sustained VA and progressive LV dysfunction. In these two studies, all young athletes except one were excluded or restricted from competitive sports during the follow-up assessments.

**Figure 7:**
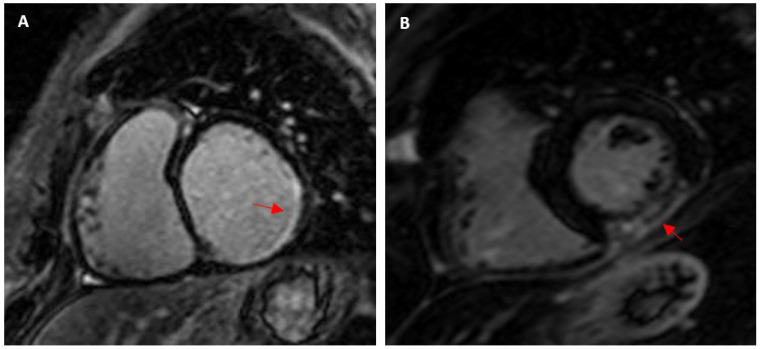
(**A**) Subendocardial LGE in the RCA territory in a 60-year-old asymptomatic male cyclist; and (**B**) Transmural LGE in a 76-year-old male marathon runner presenting with anterior T-wave inversion on electrocardiogram (possible links to coronary ischaemia, focal emboli, or coronary spasm).

Ischaemic patterns in veteran athletes may result from subclinical myocardial infarction [59] which warrants appropriate investigation and follow-up assessments (Figure 7). An increased risk of adverse outcomes has been seen in veteran marathon runners with MF and higher coronary artery calcification scores [60]. In this study, higher coronary event rates at the six-year follow-up were seen in those runners with MF and coronary calcification (57% vs. 8%) than those without MF.

### 4.5. Limitations

This review has several limitations. The studies reviewed are based on small numbers of athletes, typically healthy males. Data on female endurance athletes remain limited. The possibility of selection bias could not be excluded due to the different recruitment strategies and inclusion/exclusion criteria employed in the studies; e.g., several studies recruited athletes who were from local communities or who were self-referred. Not all athletes or controls could be guaranteed to be without cardiovascular disease, as not all underwent physical examinations or testing prior to the studies, e.g., the self-reporting of comorbidities or prior risk factors. The definition and characterisation of exercise history, athletic groups, and myocardial fibrosis may vary between studies and affect the interpretation of the data. These categories can be poorly defined and not well quantified, particularly when using self-reporting methods. This may make it somewhat difficult to accurately account for exercise history (intensity, duration, and mode) or classify an athlete or a control in studies, e.g., recreational vs. professional. Using common terminology to describe MF or LGE patterns and locations may help to facilitate a more accurate and reproducible interpretation of the studies involved, e.g., minor vs. major, patchy or spotty, and mid- or intramyocardial. Lastly, a limited number of studies in this review utilised T1 mapping to assess myocardial composition in athletes; thus, more extensive population data are needed.

## 5. Conclusions

This review highlights several factors that may predict the development of MF in athletes, with speculative insights into the associated pathophysiological processes. In most instances, an increase in cardiac remodelling and MF was associated with intense long-term endurance exercise. The patterns and extent of MF appear to vary according to the athlete’s age and sex. Although few studies on female athletes are available, women seem to be less strongly affected by MF than male athletes, which may explain the lower incidence of adverse cardiac events. MF should be assessed in clinical contexts, and the assessment of at-risk athletes is essential due to the risk of VA and SCD. Minor MF seems to be of little clinical significance; however, major MF should prompt further evaluation and follow-up assessments regarding potential cardiac disease, particularly in young athletes. The absence of extensive MF (>20% LGE) or exercise-induced arrhythmias may allow athletes to participate in most competitive sports. Additional longitudinal studies using standardized definitions for athletic groups, exercise history, and MF assessment are required in larger athletic cohorts to explore the underlying pathophysiological mechanisms of MF in athletes. Further research addressing research gaps involving more diverse athletic populations including female athletes and those from different ethnic backgrounds and long-term cardiovascular outcomes of athletes with MF are required.

## Figures and Tables

**Table 1:** Characteristics of myocardial fibrosis in athletic populations using CMR.

	Type of Sport		Athletes		CMR Findings			
Study	Athlete Group	Exercise Exposure	Age (y), Mean ± SD	Sex (%) BSA (m^2^)	LGE	Pattern/Location	T1 (ms)	ECV (%)
Zaidi et al. (2017) [13]	170 Master endurance	-	54.4 ± 8.5	M: 71 F: 29	69/170 (40.6%)	-	-	-
Verwijs et al. (2022) [14] 1.5 T, 3 T	210 Elite international, national, Olympic: 38 road cycling, 28 field hockey, 27 water polo, 21 soccer, 18 rowing, 13 swimming, 12 track cycling, 10 tennis, 10 sailing	-	28 ± 7	M: 66 2 ± 0.2 F: 34	M: 64/138 (46.4%) F: 20/72 (27.8%) Total: 84/210 (40%)	M: 64 RV insertion points F: 20 RV insertion points	959 ± 77 LGE+ 956 ± 24 LGE- 960 ± 96	24 ± 2 LGE+ 24 ± 2 LGE- 25 ± 2
Domenech Ximenos et al. (2020) [19] 1.5, 3 T	93 Triathlon	>12 h/wk training during last 5 y	35.7 ± 5.8	M: 53 1.91 ± 0.13 F: 47 1.63 ± 0.1	M: 17/49 (34.7%) F: 18/44 (40.9%) Total: 35/93 (37.6%)	RV insertion points	-	26 ± 2.3% LGE+ 27.1 ± 2.2 LGE- 25.2 ± 2.1
Banks et al. (2020) [15] 3 T	72 24 endurance runners, 20 cycling, 28 triathletes	10 y of competition, 7.6 ± 4.5 h/wk vigorous exercise	53 ± 5	M: 74 1.9 ± 0.1 F: 26 1.6 ± 0.2	M: 18/53 (34%) F: 5/16 (31%) Total: 23/69 (33%)	M: 12 RV insertion points, 2 ischaemic, 4 nonischaemic F: 5 RV insertion points	M: 1164 ± 36 F: 1190 ± 23 Total: 1169 ± 35	M: 22.1 ± 3.3 F: 24.2 ± 3.9 Total: 22.6 ± 3.5
Malek et al. (2019) [16] 3 T	30 Active healthy ultramarathon runners	Median 9 y running with frequent competitions	40.9 ± 6.6	M: 100	M: 8/30 (27%)	Nonischaemic: 5 RV insertion point, 3 septum or inferolateral wall	1200 ± 59	26.1 ± 2.9
Wilson et al. (2011) [20] 1.5 T	29 12 lifelong veteran endurance and 17 young endurance: marathon, ultramarathon, ironman, triathlon	Veteran: 43 ± 6 y of competitive training Young: 18 ± 7 y of competitive training	57 ± 6 31 ± 5	M: 100 1.96 ± 0.14 2 ± 0.14	Veteran: 6/12 (50%) Young: 0/17 Total: 6/29 (20.7%)	1 CAD pattern: subendocardial septal and lateral wall infarction pattern 5 non-CAD pattern: 1 subepicardial lateral wall (myocarditis), 4 junctional: basal and mid insertion point, inferior insertion point and mid/apical, inferior mid/apical insertion point, inferior insertion point	-	-
Sanchis-Gomar et al. (2016) [21] 3 T	53 Highly trained endurance: 11 elite and 42 sub-elite cyclists, runners	Elite: 29 ± 9 y training, 10.6 ± 3.1 h/wk Sub-elite: 24 ± 9 y training, 10.6 ± 4.2 h/wk	54 ± 4 (elite) 55 ± 9 (sub-elite)	M: 100	2/10 (20%)	Nonischaemic pattern, intra-myocardial LV lateral wall, basal inferolateral LV wall	-	-
Andresen et al. (2022) [22] 3 T	27 Healthy elite endurance athletes	379 ± 161 h/y exercise duration, 9.2 ± 0.9 MET	41 ± 9	M: 100	5/27 (18.5%)	-	1214 ± 24 (septal) LGE+ 1220 ± 4 LGE- 1212 ± 27	22.5 ± 3.1 (septal) LGE+ 22 ± 1.2 LGE- 22.7 ± 3.4
Altaha et al. (2016) [17] 3 T	33 Sub-elite endurance: 10 runners, 12 cyclists, 10 triathletes	>10 y of exercise, 4.8 ± 2.5 h/wk exercise	55 ± 5.6	M: 76 F: 24	M: 4/25 (16%) F: 1/8 (12.5%) Total: 5/33 (15.2%)	Non-specific RV inferior hinge-point	-	-
La Gerche et al. (2012) [18] 1.5 T	40 7 marathon runners, 11 endurance triathletes, 9 alpine cyclists, 13 ultra-triathletes	10 ± 9 years training, 16.3 ± 5.1 h/wk intense training	37 ± 8	M: 90 F: 10	5/39 (12.8%)	Interventricular septum, frequently in the RV attachment	-	-
Bosscher et al. (2020) [23] 3 T	231 Elite endurance athletes	-	18 ± 2 (young) 38 ± 5 (middle-aged)	M: 79 F: 21	M: 27/187 (14.4%) F: 1/50 (2%) Total: 28 (12.5%)	M: 24 RV insertion points, 3 subepicardial LV lateral wall F: 1 RV insertion points	-	-
Breuckmann et al. (2009) [24] 1.5 T	102 Marathon runners	≥5 marathons in ≤3 y	57 ± 6	M: 100	12/102 (11.8%)	5: subendocardial layer typical myocardial infarction (10 LAD, 1 LCA, 3 RCA segments) 7: mid-myocardial patchy nonischaemic pattern (3 LAD, 5 LCA, 9 RCA segments)	-	-
Tahir et al. (2018) [11] 1.5 T	83 Triathletes	12.6 y competitions, >10 h/wk training	43 ± 10	M: 65 1.98 ± 0.12 F: 35 1.73 ± 0.12	M: 9/54 (16.7%) F: 0/29 Total: 9/83 (10.8%)	Nonischaemic: 5: subepicardial (myocarditis)–inferolateral LV wall, 2: posterior RV insertion points, 1: transmural	M: 990 ± 28 F: 1015 ± 25 M: LGE+ 1005 ± 32 M: LGE- 987 ± 27	M: 24.8 ± 2.2 F: 27.8 ± 1.9 M: LGE+ 26.3 ± 1.8 M: LGE- 24.4 ± 2.2
Merghani et al. (2017) [12] 1.5 T	152 Master cyclists, runners	M: 33.4 ± 12.9 y endurance exercise, 7.5 ± 3.8 h/wk F: 26.1 ± 10.9 y endurance exercise, 7.7 ± 2.9 h/wk	54.4 ± 8.5	M: 70 1.9 ± 0.12 F: 30 1.62 ± 0.12	M: 15/106 (14.2%) F: 1/46 (2.2%) Total: 16/152 (10.5%)	M: 7 subendocardial LGE consistent with myocardial infarction, 5 midmyocardial, 3 epicardial distribution F: 1 subendocardial LGE	-	-
Pujadas et al. (2018) [25] 1.5 T	34 Healthy endurance veterans: marathons	28.06 ± 10.84 y training, 9.38 ± 3.52 h/wk, still in regular training	48.17 ± 7.4	M: 100 1.8 ± 0.11	3/34 (8.8%)	Nonischaemic: mesocardial in septal–apical wall, subepicardial inferior apical wall, mesocardial lateral wall	943.6 ± 53 (septal)	25 ± 2% (septal)
Karlstedt et al. (2012**)** [26] 1.5 T	25 Healthy marathon runners	≥3 marathons in the past 2 y, 47 ± 7 miles/wk training	55 ± 4	M: 84 F: 16	2/25 (8%)	Subendocardial distribution of LV anterior wall (before running marathon), with evidence of obstructive LAD artery disease	-	-
Swoboda et al. (2016) [27] 3 T	40 Competitive athletes: 11 runners, 13 triathletes, 16 cyclists	>6 h/wk training	<45 y	-	2/40 (5%)	Subepicardial lateral in a myocarditis pattern	1182.7 ± 42.4	22.7 ± 3.3
McDiarmid et al. (2016) [28] 3 T	30 Endurance: 7 runners, 11 cyclists, 12 triathletes	Regional, national, or international level >6 h/wk training	31.7 ± 7.7	M: 100	1/30 (3.3%)	Nonischaemic (post myocarditis pattern)	1178 ± 32	22.5 ± 2.6
Bohm et al. (2016) [29] 1.5 T	33 16 former elite master endurance athletes: marathon, triathlons, ironman, rowing, cycling	29 ± 8 y training history, 16.7 ± 4.4 h/wk training	47 ± 8	M: 100 1.96 ± 0.1	1/33 (3%)	Nonischaemic, subepicardial LV posteroinferior region (most likely due to former pericarditis)	-	-
Mangold et al. (2013) [30] 1.5 T	95 39 long-distance runners, 8 cyclists, 34 triathletes, 13 handball players, 1 speed skater	13.1 ± 4.2 h/wk for ≥2 y M: 13.1 ± 4.5 h/wk (5–30) F: 12.8 ± 3 h/wk (7–20)	35.2 ± 11.4	M: 77 1.91 ± 0.13 F: 23 1.7 ± 0.2	M: 1/73 (1.3%) F: 1/22 (4.4%) Total: 2/95 (2.1%)	Nonischaemic, post myocarditis pattern (spot-shaped), disseminated and intramural	-	-
Androulakis et al. (2022) [31]	61 Endurance sport: 40	12.5 ± 3.3 h/wk	27.9 ± 6.7	M: 80 1.99 ± 0.2 F: 20	60	Minor MF: 28 insertion points Major MF: 18 mid-myocarial, 10 subepicardial, 4 subepicardial	-	-

CAD: coronary artery disease, CMR: cardiac magnetic resonance, ECV: extracellular volume, F: female, h: hours, LAD: left anterior descending artery, LCA: left circumflex artery, LGE: late gadolinium enhancement, LV: left ventricle, M: male, MET: metabolic equivalent of task, RCA: right coronary artery, RV: right ventricle, SD: standard deviation, T: Tesla, wk: week, y: years. Studies arranged from highest to lowest MF prevalence.

**Table 2:** T1 mapping and ECV quantification in athletic populations using CMR.

	Athletes				
CMR Findings	All	Male	Female	Young	Veteran
1.5 T (3 studies)	(n = 145)	(n = 88)	(n = 29)	(n = 28)	(n = 117)
T1 (ms)	990.9 ± 32 *	966.8 ± 40	1015 ± 25	-	990.9 ± 32
ECV (%)	26.2 ± 2.1	24.9 ± 2.1	27.8 ± 1.9	26 ± 2.3	26.3 ± 2
LGE + (1.5 T) (2 studies)		(n = 22)			
T1 (ms)	-	1005 ± 32	(n = 9)	-	-
ECV (%)	-	26.7 ± 2	(n = 22)	-	-
LGE − (1.5 T)		(n = 60)			
T1 (ms)	-	987 ± 27	(n = 45)	-	-
ECV (%)	-	24.8 ± 2.1	(n = 60)	-	-
3 T (5 studies)	(n = 177)	(n = 95)	(n = 12)	(n = 30)	(n = 107)
T1 (ms)	1185.8 ± 34	1192.7 ± 40	1190 ± 23	1178 ± 32	1191.3 ± 31
ECV (%)	23.2 ± 3.3	23.6 ± 3.1	24.2 ± 3.9	22.5 ± 2.6	23.9 ± 3.5
LGE + (3 T) (1 study)		(n = 5)			
T1 (ms)	-	1220 ± 4	-	-	-
ECV (%)	-	22 ± 1.2	-	-	-
LGE − (3 T)		(n = 22)			
T1 (ms)	-	1212 ± 27	-	-	-
ECV (%)	-	22.7 ± 3.4	-	-	-
	Controls				
1.5 T (2 studies)	(n = 48)	(n = 34)	(n = 14)		(n = 48)
T1 (ms)	1029 ± 27	999.1 ± 32	1059 ± 22	-	1029 ± 27
ECV (%)	25.95 ± 2.9	23 ± 2.5	28.9 ± 3.3	-	25.95 ± 2.9
3 T (5 studies)	(n = 97)	(n = 35)	(n = 7)	(n = 20)	(n = 42)
T1 (ms)	1207.5 ± 32 ^	1221.7 ± 35	1197 ± 22	1202 ± 33	1209.3 ± 28
ECV (%)	22.84 ± 2.6	23.7 ± 2.6	20.4 ± 2.8	24.5 ± 2.2	22 ± 2.7

CMR: cardiac magnetic resonance, ECV: extracellular volume, LGE: late gadolinium enhancement. * T1 not reported in 28 athletes, ^ T1 not reported in 35 controls. Study 14: 210 athletes not included in analysis (combined 1.5/3T data).

## Data Availability

The original contributions presented in the study are included in the article/Supplementary Materials; further inquiries can be directed to the corresponding authors.

## Supplementary Materials

The following supporting information can be downloaded at: https://www.mdpi.com/article/10.3390/jcm13154536/s1.

## References

[1] Pelliccia A., Sharma S., Gati S., Bäck M., Börjesson M., Caselli S., Collet J.-P., Corrado D., Drezner J.A., Halle M. (2021). 2020 ESC Guidelines on sports cardiology and exercise in patients with cardiovascular disease. Eur. Heart J..

[2] Małek Ł.A., Bucciarelli-Ducci C. (2020). Myocardial fibrosis in athletes—Current perspective. Clin. Cardiol..

[3] van de Schoor F.R., Aengevaeren V.L., Hopman M.T., Oxborough D.L., George K.P., Thompson P.D., Eijsvogels T.M. (2016). Myocardial Fibrosis in Athletes. Mayo Clin. Proc..

[4] Bing R., Dweck M.R. (2019). Myocardial fibrosis: Why image, how to image and clinical implications. Heart.

[5] Androulakis E., Mouselimis D., Tsarouchas A., Antonopoulos A., Bakogiannis C., Papagkikas P., Vlachopoulos C. (2021). The Role of Cardiovascular Magnetic Resonance Imaging in the Assessment of Myocardial Fibrosis in Young and Veteran Athletes: Insights from a Meta-Analysis. Front. Cardiovasc. Med..

[6] Zhang C.-D., Xu S.-L., Wang X.-Y., Tao L.-Y., Zhao W., Gao W. (2020). Prevalence of Myocardial Fibrosis in Intensive Endurance Training Athletes: A Systematic Review and Meta-Analysis. Front. Cardiovasc. Med..

[7] Androulakis E., Swoboda P.P. (2018). The Role of Cardiovascular Magnetic Resonance in Sports Cardiology; Current Utility and Future Perspectives. Curr. Treat. Options Cardiovasc. Med..

[8] Cummings K.W., Bhalla S., Javidan-Nejad C., Bierhals A.J., Gutierrez F.R., Woodard P.K. (2009). A pattern-based approach to assessment of delayed enhancement in nonischemic cardiomyopathy at MR imaging. Radiographics.

[9] Maestrini V., Merghani A., Rosmini S., Cox A., Bulluck H., Culotta V., Cheang M., Fontana M., A Treibel T., Abdel-Gadir A. (2016). CMR findings in high endurance veteran athletes-a 247 subject study. J. Cardiovasc. Magn. Reson..

[10] Page M.J., McKenzie J.E., Bossuyt P.M., Boutron I., Hoffmann T.C., Mulrow C.D., Shamseer L., Tetzlaff J.M., Akl E.A., Brennan S.E. (2021). The PRISMA 2020 statement: An updated guideline for reporting systematic reviews. BMJ.

[11] Tahir E., Starekova J., Muellerleile K., von Stritzky A., Münch J., Avanesov M., Weinrich J.M., Stehning C., Bohnen S., Radunski U.K. (2018). Myocardial Fibrosis in Competitive Triathletes Detected by Contrast-Enhanced CMR Correlates with Exercise-Induced Hypertension and Competition History. JACC Cardiovasc. Imaging.

[12] Merghani A., Maestrini V., Rosmini S., Cox A.T., Dhutia H., Bastiaenan R., David S., Yeo T.J., Narain R., Malhotra A. (2017). Prevalence of Subclinical Coronary Artery Disease in Masters Endurance Athletes with a Low Atherosclerotic Risk Profile. Circulation.

[13] Zaidi A., Merghani A., Maestrini V., Rosmini S., Schofield R., Papadakis M., Manisty C., Moon J., Sharma S. (2017). P3990Exercise-induced arrhythmogenic right ventricular remodeling in master endurance athletes. Eur. Heart J..

[14] Verwijs S.M., Van Hattum J.C., Spies J.L., Boekholdt S., Planken, Groenink M., Van Randen A., Van Luijk R., Berg-Faaij A.V.D., Bakermans A. (2022). Late gadolinium enhancement of the hinge point is a common finding in asymptomatic ELITE athletes. Eur. J. Prev. Cardiol..

[15] Banks L., Altaha M.A., Yan A.T., Dorian P., Konieczny K., Deva D.P., LA Gerche A., Akhavein F., Bentley R.F., Connelly K.A. (2020). Left Ventricular Fibrosis in Middle-Age Athletes and Physically Active Adults. Med. Sci. Sports Exerc..

[16] Małek Ł.A., Barczuk-Falęcka M., Werys K., Czajkowska A., Mróz A., Witek K., Burrage M., Bakalarski W., Nowicki D., Roik D. (2019). Cardiovascular magnetic resonance with parametric mapping in long-term ultra-marathon runners. Eur. J. Radiol..

[17] Altaha M.A., Connelly K., Yan A.T., Banks L., Dorian P., Goodman J. (2016). Prevalence of late gadolinium enhancement in middle-aged, sub-elite athletes. Can. J. Cardiol..

[18] La Gerche A., Burns A.T., Mooney D.J., Inder W.J., Taylor A.J., Bogaert J., MacIsaac A.I., Heidbüchel H., Prior D.L. (2012). Exercise-induced right ventricular dysfunction and structural remodelling in endurance athletes. Eur. Heart J..

[19] Domenech-Ximenos B., la Garza M.S.-D., Prat-González S., Sepúlveda-Martínez A., Crispi F., Duran-Fernandez K., Perea R.J., Bijnens B., Sitges M. (2020). Prevalence and pattern of cardiovascular magnetic resonance late gadolinium enhancement in highly trained endurance athletes. J. Cardiovasc. Magn. Reson..

[20] Wilson M., O’Hanlon R., Prasad S., Deighan A., MacMillan P., Oxborough D., Godfrey R., Smith G., Maceira A., Sharma S. (2011). Diverse patterns of myocardial fibrosis in lifelong, veteran endurance athletes. J. Appl. Physiol..

[21] Sanchis-Gomar F., López-Ramón M., Alis R., Garatachea N., Pareja-Galeano H., Santos-Lozano A., Catalán P., Sansoni V., Perego S., Lombardi G. (2016). No evidence of adverse cardiac remodeling in former elite endurance athletes. Int. J. Cardiol..

[22] Andresen K., Klaeboe L.G., Lie Ø.H., Broch K., Kvaslerud A., Bosse G., Hopp E., Haugaa K., Edvardsen T. (2022). No signs of diffuse myocardial fibrosis by T1 mapping in male elite endurance athletes. Eur. Heart J.-Cardiovasc. Imaging.

[23] De Bosscher R., Claeys M., Dausin C., Goetschalckx K., Bogaert J., Van De Heyning C., Ghekiere O., Herbots L., Claus P., Kalman J. (2020). Hinge point fibrosis in athletes is not associated with structural, functional or electrical consequences: A comparison between young and middle-aged elite endurance athletes. Eur. Heart J..

[24] Breuckmann F., Möhlenkamp S., Nassenstein K., Lehmann N., Ladd S., Schmermund A., Sievers B., Schlosser T., Jöckel K.-H., Heusch G. (2009). Myocardial late gadolinium enhancement: Prevalence, pattern, and prognostic relevance in marathon runners. Radiology.

[25] Pujadas S., Doñate M., Li C.-H., Merchan S., Cabanillas A., Alomar X., Pons-Llado G., Serra-Grima R., Carreras F. (2018). Myocardial remodelling and tissue characterisation by cardiovascular magnetic resonance (CMR) in endurance athletes. BMJ Open Sport Exerc. Med..

[26] Karlstedt E., Chelvanathan A., Da Silva M., Cleverley K., Kumar K., Bhullar N., Lytwyn M., Bohonis S., Oomah S., Nepomuceno R. (2012). The impact of repeated marathon running on cardiovascular function in the aging population. J. Cardiovasc. Magn. Reson..

[27] Swoboda P.P., McDiarmid A.K., Erhayiem B., Broadbent D.A., Dobson L.E., Garg P., Ferguson C., Page S.P., Greenwood J.P., Plein S. (2016). Assessing Myocardial Extracellular Volume by T1 Mapping to Distinguish Hypertrophic Cardiomyopathy from Athlete’s Heart. J. Am. Coll. Cardiol..

[28] McDiarmid A.K., Swoboda P.P., Erhayiem B., Lancaster R.E., Lyall G.K., Broadbent D.A., Dobson L.E., Musa T.A., Ripley D.P., Garg P. (2016). Athletic Cardiac Adaptation in Males Is a Consequence of Elevated Myocyte Mass. Circ. Cardiovasc. Imaging.

[29] Bohm P., Schneider G., Linneweber L., Rentzsch A., Krämer N., Abdul-Khaliq H., Kindermann W., Meyer T., Scharhag J. (2016). Right and Left Ventricular Function and Mass in Male Elite Master Athletes: A Controlled Contrast-Enhanced Cardiovascular Magnetic Resonance Study. Circulation.

[30] Mangold S., Kramer U., Franzen E., Erz G., Bretschneider C., Seeger A., Claussen C.D., Niess A.M., Burgstahler C. (2013). Detection of cardiovascular disease in elite athletes using cardiac magnetic resonance imaging. Rofo-Fortschritte Geb. Rontgenstrahlen Bild. Verfahr..

[31] Androulakis E., Papatheodorou S., Merghani A., Sharma S., Papadakis M. (2022). Patterns and clinical significance of non-specific myocardial fibrosis; Evidence from a cohort of young competitive athletes referred to a tertiary referral centre. Eur. J. Prev. Cardiol..

[32] Franzen E., Mangold S., Erz G., Claussen C.D., Niess A.M., Kramer U., Burgstahler C. (2013). Comparison of morphological and functional adaptations of the heart in highly trained triathletes and long-distance runners using cardiac magnetic resonance imaging. Heart Vessel..

[33] Abdullah S.M., Barkley K.W., Bhella P.S., Hastings J.L., Matulevicius S., Fujimoto N., Shibata S., Carrick-Ranson G., Palmer M.D., Gandhi N. (2016). Lifelong Physical Activity Regardless of Dose Is Not Associated with Myocardial Fibrosis. Circ. Cardiovasc. Imaging.

[34] Turkbey E.B., Nacif M.S., Guo M., McClelland R.L., Teixeira P.B.R.P., Bild D.E., Barr R.G., Shea S., Post W., Burke G. (2015). Prevalence and Correlates of Myocardial Scar in a US Cohort. JAMA.

[35] Barbier C.E., Bjerner T., Johansson L., Lind L., Ahlström H. (2006). Myocardial scars more frequent than expected: Magnetic resonance imaging detects potential risk group. J. Am. Coll. Cardiol..

[36] Schelbert E.B., Cao J.J., Sigurdsson S., Aspelund T., Kellman P., Aletras A.H., Dyke C.K., Thorgeirsson G., Eiriksdottir G., Launer L.J. (2012). Prevalence and prognosis of unrecognized myocardial infarction determined by cardiac magnetic resonance in older adults. JAMA.

[37] Baggish A.L. (2018). Focal Fibrosis in the Endurance Athlete’s Heart Running Scarred or Running Scared?. JACC: Cardiovasc. Imaging.

[38] Benito B., Gay-Jordi G., Serrano-Mollar A., Guasch E., Shi Y., Tardif J.-C., Brugada J., Nattel S., Mont L. (2011). Cardiac arrhythmogenic remodeling in a rat model of long-term intensive exercise training. Circulation.

[39] La Gerche A., Heidbüchel H., Burns A.T., Mooney D.J., Taylor A.J., Pfluger H.B., Inder W.J., Macisaac A.I., Prior D.L. (2011). Disproportionate exercise load and remodeling of the athlete’s right ventricle. Med. Sci. Sports Exerc..

[40] Castelletti S., Gati S. (2021). The Female Athlete’s Heart: Overview and Management of Cardiovascular Diseases. Eur. Cardiol. Rev..

[41] D’Ascenzi F., Biella F., Lemme E., Maestrini V., Di Giacinto B., Pelliccia A. (2020). Female Athlete’s Heart: Sex Effects on Electrical and Structural Remodeling. Circ. Cardiovasc. Imaging.

[42] Graziano F., Juhasz V., Brunetti G., Cipriani A., Szabo L., Merkely B., Corrado D., D’ascenzi F., Vago H., Zorzi A. (2022). May Strenuous Endurance Sports Activity Damage the Cardiovascular System of Healthy Athletes? A Narrative Review. J. Cardiovasc. Dev. Dis..

[43] Shanbhag S.M., Greve A.M., Aspelund T., Schelbert E.B., Cao J.J., Danielsen R., Þorgeirsson G., Sigurðsson S., Eiríksdóttir G., Harris T.B. (2019). Prevalence and prognosis of ischaemic and non-ischaemic myocardial fibrosis in older adults. Eur. Heart J..

[44] Coronado M.J., Bruno K.A., Blauwet L.A., Tschöpe C., Cunningham M.W., Pankuweit S., van Linthout S., Jeon E., McNamara D.M., Krejčí J. (2019). Elevated Sera sST2 Is Associated with Heart Failure in Men ≤50 Years Old with Myocarditis. J. Am. Heart Assoc..

[45] Cabinian A.E., Kiel R.J., Smith F., Ho K.L., Khatib R., Reyes M.P. (1990). Modification of exercise-aggravated coxsackievirus B3 murine myocarditis by T lymphocyte suppression in an inbred model. J. Lab. Clin. Med..

[46] Möhlenkamp S., Lehmann N., Breuckmann F., Bröcker-Preuss M., Nassenstein K., Halle M., Budde T., Mann K., Barkhausen J., Heusch G. (2008). Marathon Study Investigators; Heinz Nixdorf Recall Study Investigators. Running: The risk of coronary events: Prevalence and prognostic relevance of coronary atherosclerosis in marathon runners. Eur. Heart J..

[47] Zambrano A., Tintut Y., Demer L.L., Hsu J.J. (2023). Potential mechanisms linking high-volume exercise with coronary artery calcification. Heart.

[48] Aengevaeren V.L., Mosterd A., Sharma S., Prakken N.H., Möhlenkamp S., Thompson P.D., Velthuis B.K., Eijsvogels T.M. (2020). Exercise and Coronary Atherosclerosis: Observations, Explanations, Relevance, and Clinical Management. Circulation.

[49] Rao S.J., Shah A.B. (2022). Exercise and the Female Heart. Clin. Ther..

[50] Di Marco A., Brown P.F., Bradley J., Nucifora G., Anguera I., A Miller C., Schmitt M. (2022). Extracellular volume fraction improves risk-stratification for ventricular arrhythmias and sudden death in non-ischaemic cardiomyopathy. Eur. Heart J.-Cardiovasc. Imaging.

[51] Zhu L., Wang Y., Zhao S., Lu M. (2022). Detection of myocardial fibrosis: Where we stand. Front. Cardiovasc. Med..

[52] Haaf P., Garg P., Messroghli D.R., Broadbent D.A., Greenwood J.P., Plein S. (2016). Cardiac T1 Mapping and Extracellular Volume (ECV) in clinical practice: A comprehensive review. J. Cardiovasc. Magn. Reson..

[53] Szabo L., Brunetti G., Cipriani A., Juhasz V., Graziano F., Hirschberg K., Dohy Z., Balla D., Drobni Z., Marra M.P. (2022). Certainties and Uncertainties of Cardiac Magnetic Resonance Imaging in Athletes. J. Cardiovasc. Dev. Dis..

[54] Grigoratos C., Pantano A., Meschisi M., Gaeta R., Ait-Ali L., Barison A., Todiere G., Festa P., Sinagra G., Aquaro G.D. (2020). Clinical importance of late gadolinium enhancement at right ventricular insertion points in otherwise normal hearts. Int. J. Cardiovasc. Imaging.

[55] Crescenzi C., Zorzi A., Vessella T., Martino A., Panattoni G., Cipriani A., De Lazzari M., Marra M.P., Fusco A., Sciarra L. (2021). Predictors of Left Ventricular Scar Using Cardiac Magnetic Resonance in Athletes with Apparently Idiopathic Ventricular Arrhythmias. J. Am. Heart Assoc..

[56] Zorzi A., Marra M.P., Rigato I., De Lazzari M., Susana A., Niero A., Pilichou K., Migliore F., Rizzo S., Giorgi B. (2016). Nonischemic Left Ventricular Scar as a Substrate of Life-Threatening Ventricular Arrhythmias and Sudden Cardiac Death in Competitive Athletes. Circ. Arrhythmia Electrophysiol..

[57] Brunetti G., Graziano F., Cavigli L., Cipriani A., D’ascenzi F., Bauce B., Pilichou K., Marra M.P., Corrado D., Zorzi A. (2022). Reproducibility of ventricular arrhythmias at exercise testing for prediction of non-ischemic left ventricular scar in athletes. Eur. J. Prev. Cardiol..

[58] Schnell F., Claessen G., La Gerche A., Bogaert J., Lentz P.-A., Claus P., Mabo P., Carré F., Heidbuchel H. (2016). Subepicardial delayed gadolinium enhancement in asymptomatic athletes: Let sleeping dogs lie?. Br. J. Sports Med..

[59] Parry-Williams G., Gati S., Sharma S. (2021). The heart of the ageing endurance athlete: The role of chronic coronary stress. Eur. Heart J..

[60] Möhlenkamp S., Leineweber K., Lehmann N., Braun S., Roggenbuck U., Perrey M., Broecker-Preuss M., Budde T., Halle M., Mann K. (2014). Coronary atherosclerosis burden, but not transient troponin elevation, predicts long-term outcome in recreational marathon runners. Basic Res. Cardiol..

