# Supplementary material for: Myocardial Fibrosis in Young and Veteran Athletes: Evidence from a Systematic Review of the Current Literature
Source: J Clin Med. 2024 Aug 2;13(15):4536. doi: 10.3390/jcm13154536 (PMC11313657; doi:10.3390/jcm13154536)
Supplement: Supplementary file 1 [file jcm-13-04536-s001.zip › jcm-3113261-supplementary.pdf]

Supplementary Material

Supplementary Table S1. Risk of bias assessment via the Newcastle Ottawa Scale for Cohort Studies.

	**Selection**			**Comparability**	**Outcome**		
**Study**	Representativeness of athletes (★)	Selection of controls (★)	Ascertainment of athlete status (★)	(★★)	Assessment of CMR findings (★)	Was duration of intense athleticism enough for outcomes to occur (★)	Total (7★)
Zaidi et al. 2017 [13]	★	-	-	★	★	-	3
Verwijs et al. 2022 [14]	★	-	★	★	★	-	4
Domenench-Ximenos et al. 2020 [19]	★	-	★	★★	★	★	6
Banks et al. 2020 [15]	★	★	★	-	★	★	5
Malek et al. 2019 [16]	★	-	★	★★	-	-	4
Wilson et al. 2011 [20]	★	★	★	★	★	★	6
Sanchis-Gomar et al. 2016 [21]	★	-	-	★★	★	★	5
Andresen et al. 2022 [22]	★	★	★	★	★	★	6
Altaha et al. 2016 [17]	★	-	★	★	★	-	4
La Gerche et al. 2012 [18]	★	-	★	★	★	★	5
Bosscher et al. 2020 [23]	★	-	★	★	★	★	5
Breuckman et al. 2009 [24]	★	★	★	★	-	★	5
Tahir et al. 2018 [11]	★	-	★	★	★	★	5
Merghani et al. 2017 [12]	★	★	★	-	★	★	5
Pujadas et al. 2018 [25]	★	-	★	★★	-	★	5
Karlstedt et al 2012 [26]	★	-	-	★	★	★	4
Swoboda et al. 2016 [27]	★	-	★	★★	-	-	4
Mc Diarmid et al. 2016 [28]	★	-	★	★	★	-	4
Bohm et al. 2016 [29]	★	-	-	★★	-	★	4
Mangold et al. 2013 [30]	★	-	★	★	★	★	5
Androulakis et al. 2022 [31]	★	★	-	★	★	★	5

1 Search strategy

The search process was conducted in accordance with the Preferred Reporting Items for Systematic Reviews and Meta-Analyses (PRISMA) guidelines. A systematic search of relevant studies published up to 18th February 2023 was performed in PubMed/MEDLINE and Google Scholer. The keywords were related to athlete ("athlete", "athlete's heart"), myocardial fibrosis ("myocardial fibrosis", "fibrosis", "scar"), cardiac magnetic resonance imaging, late gadolinium enhancement ("late gadolinium enhancement", "delayed gadolinium enhancement"), T1 and extracellular volume ("T1 mapping", "native T1 values", "extracellular volume") were used as search terms. R.A. and E.M. independently selected which of the search results fulfilled inclusion criteria. In cases of disagreement, it was predetermined that M.P. was going to serve as an arbitrator.

1.1 Inclusion criteria

Publications reporting on outcomes of studies having evaluated one or more of the following parameters in high-performance athletes:

- The presence of late gadolinium enhancement
- Native T1 values
- Extracellular volume

Only studies recruiting athletes trained in endurance sports were included, which was defined as having certain competition experience or a long period of regular high intensity endurance training [6]. Veteran athletes were defined as a mean age >40 years [5]. Only studies reported in English were evaluated for inclusion.

1.2 Exclusion criteria

Studies where either athletes or controls had been included on the basis of having symptoms or signs of cardiac pathology (e.g. premature ventricular beats) were excluded from the systematic review. When multiple studies included results reported on data from the same research group, only one was kept, unless it is explicitly stated that there is no overlap.

1.3 Data extraction

The following data was extracted from each study: Author, MRI scanner, publication year, athlete/control participant numbers, athlete/control age, sex, body surface area, and exercise exposure (e.g. types of sports, activity level etc.). Qualitative LGE data were also extracted, with observed prevalence for whole-heart as well as regional-specific (patterns) LGE data (e.g. insertion point, subepicardial LGE etc.) being recorded for each study. Finally, T1 and ECV values for athletes and controls were also extracted, if available. The synthesis of LGE was performed on the basis of proportions in the athlete and control group (including male, female, young, and veteran where possible), while native T1 (measured in msec) and ECV (reported as a percentage) were synthesized between studies and compared between groups as mean difference. Extracted data was stored in Word file or Microsoft Excel datasheets.

1.4 Quality assessment

To evaluate the methodological quality of the selected studies, assessment was performed using the Newcastle-Ottawa quality assessment scale (NOS) for cohort studies (**Supplementary Table 1**). The NOS assessment consisted of three domains: selection, comparability, and outcome.

PRISMA 2020 Checklist

Section and Topic	Item #	Checklist item	Location where item is reported
**TITLE**			
Title	1	Identify the report as a systematic review.	1
**ABSTRACT**			
Abstract	2	See the PRISMA 2020 for Abstracts checklist.	1
**INTRODUCTION**			
Rationale	3	Describe the rationale for the review in the context of existing knowledge.	1-2
Objectives	4	Provide an explicit statement of the objective(s) or question(s) the review addresses.	1-2
**METHODS**			
Eligibility criteria	5	Specify the inclusion and exclusion criteria for the review and how studies were grouped for the syntheses.	2-3, Supplementary Data
Information sources	6	Specify all databases, registers, websites, organisations, reference lists and other sources searched or consulted to identify studies. Specify the date when each source was last searched or consulted.	2-3
Search strategy	7	Present the full search strategies for all databases, registers and websites, including any filters and limits used.	2-3, Supplementary Data
Selection process	8	Specify the methods used to decide whether a study met the inclusion criteria of the review, including how many reviewers screened each record and each report retrieved, whether they worked independently, and if applicable, details of automation tools used in the process.	2-3, Supplementary Data
Data collection process	9	Specify the methods used to collect data from reports, including how many reviewers collected data from each report, whether they worked independently, any processes for obtaining or confirming data from study investigators, and if applicable, details of automation tools used in the process.	Supplementary Data
Data items	10a	List and define all outcomes for which data were sought. Specify whether all results that were compatible with each outcome domain in each study were sought (e.g. for all measures, time points, analyses), and if not, the methods used to decide which results to collect.	Supplementary Data
	10b	List and define all other variables for which data were sought (e.g. participant and intervention characteristics, funding sources). Describe any assumptions made about any missing or unclear information.	Supplementary Data
Study risk of bias assessment	11	Specify the methods used to assess risk of bias in the included studies, including details of the tool(s) used, how many reviewers assessed each study and whether they worked independently, and if applicable, details of automation tools used in the process.	2-3
Effect measures	12	Specify for each outcome the effect measure(s) (e.g. risk ratio, mean difference) used in the synthesis or presentation of results.	Supplementary Data
Synthesis methods	13a	Describe the processes used to decide which studies were eligible for each synthesis (e.g. tabulating the study intervention characteristics and comparing against the planned groups for each synthesis (item #5)).	2-3, Supplementary Data
	13b	Describe any methods required to prepare the data for presentation or synthesis, such as handling of missing summary statistics, or data conversions.	2-3, Supplementary Data
	13c	Describe any methods used to tabulate or visually display results of individual studies and syntheses.	Supplementary Data
	13d	Describe any methods used to synthesize results and provide a rationale for the choice(s). If meta-analysis was performed, describe the model(s), method(s) to identify the presence and extent of statistical heterogeneity, and software package(s) used.	2-3, Supplementary Data

PRISMA 2020 Checklist

Section and Topic	Item #	Checklist item	Location where item is reported
	13e	Describe any methods used to explore possible causes of heterogeneity among study results (e.g. subgroup analysis, meta-regression).	2-3
	13f	Describe any sensitivity analyses conducted to assess robustness of the synthesized results.	2-3
Reporting bias assessment	14	Describe any methods used to assess risk of bias due to missing results in a synthesis (arising from reporting biases).	2-3, Supplementary Table
Certainty assessment	15	Describe any methods used to assess certainty (or confidence) in the body of evidence for an outcome.	N/A
**RESULTS**			
Study selection	16a	Describe the results of the search and selection process, from the number of records identified in the search to the number of studies included in the review, ideally using a flow diagram.	4, Figure 1
	16b	Cite studies that might appear to meet the inclusion criteria, but which were excluded, and explain why they were excluded.	Supplementary Data
Study characteristics	17	Cite each included study and present its characteristics.	4-9
Risk of bias in studies	18	Present assessments of risk of bias for each included study.	4, Supplementary Table
Results of individual studies	19	For all outcomes, present, for each study: (a) summary statistics for each group (where appropriate) and (b) an effect estimate and its precision (e.g. confidence/credible interval), ideally using structured tables or plots.	4-9
Results of syntheses	20a	For each synthesis, briefly summarise the characteristics and risk of bias among contributing studies.	4-9
	20b	Present results of all statistical syntheses conducted. If meta-analysis was done, present for each the summary estimate and its precision (e.g. confidence/credible interval) and measures of statistical heterogeneity. If comparing groups, describe the direction of the effect.	N/A
	20c	Present results of all investigations of possible causes of heterogeneity among study results.	4-9
	20d	Present results of all sensitivity analyses conducted to assess the robustness of the synthesized results.	N/A
Reporting biases	21	Present assessments of risk of bias due to missing results (arising from reporting biases) for each synthesis assessed.	N/A
Certainty of evidence	22	Present assessments of certainty (or confidence) in the body of evidence for each outcome assessed.	N/A
**DISCUSSION**			
Discussion	23a	Provide a general interpretation of the results in the context of other evidence.	10-14
	23b	Discuss any limitations of the evidence included in the review.	15
	23c	Discuss any limitations of the review processes used.	15
	23d	Discuss implications of the results for practice, policy, and future research.	12-14
**OTHER INFORMATION**			
Registration and protocol	24a	Provide registration information for the review, including register name and registration number, or state that the review was not registered.	N/A
	24b	Indicate where the review protocol can be accessed, or state that a protocol was not prepared.	3
	24c	Describe and explain any amendments to information provided at registration or in the protocol.	NA
Support	25	Describe sources of financial or non-financial support for the review, and the role of the funders or sponsors in the review.	16

PRISMA 2020 Checklist

Section and Topic	Item #	Checklist item	Location where item is reported
Competing interests	26	Declare any competing interests of review authors.	16
Availability of data, code and other materials	27	Report which of the following are publicly available and where they can be found: template data collection forms; data extracted from included studies; data used for all analyses; analytic code; any other materials used in the review.	16

From: Page MJ, McKenzie JE, Bossuyt PM, Boutron I, Hoffmann TC, Mulrow CD, et al. The PRISMA 2020 statement: an updated guideline for reporting systematic reviews. BMJ 2021;372:n71. doi: 10.1136/bmj.n71[10].
For more information, visit: http://www.prisma-statement.org/
